# Extracts from Prof. M’Gugin’s Introductory

**Published:** 1853-12

**Authors:** 


### *Extract from Prof. M'Gugin's Introductory to the present Course of Lectures of the Iowa Medical Department.*

As a beneficent scheme, medical science seeks to alleviate the suffering and assuage the griefs and woes of our fellow men. To be the instrument, in the hands of Providence, in saving life, is to hold a position the most exalted among men. To be able to relieve suffering, when a cure is even impossible, is to fulfil a distinguished office and perform an important function in life. To smooth the bed of death and relieve the sufferer of its severe inflictions, to enable our dying fellow mortal to glide down softly to the tomb, with the usual agonies unfelt and unendured, are attributes and duties, in the discharge of which humanity will shed its grateful tears, and for which angels will weep for joy.

It is everywhere on its errand of mercy. You will find it dispensing its blessings and benefits in the "mad house," where "the mind is lost in the stormy deserts of the brain." Here by his instrumentality the wandering intellect is brought back, and reason and rationality restored. The imaginary horrors, experienced during the night of the mind, are by the skill and treatment of the physician, moral, mental, and physical, driven away and dispelled. But medical science has not only relieved the mind from the bondage of darknes, but it has loosed the chains, which ignorance and inhumanity had put upon their limbs, thereby riveting the disease and confirming the deplorable malady upon them. While Pinel was opening the prison doors of filthy cells, in which these unhappy objects were incarcerated for half a century, he not only loosed the chains that bound them, but by this very act restored the mind also to mental light and liberty. The diferent asylums of the world, erected by the philanthropy and liberality of a christian people, as monuments of their christian sympathy, are managed, conducted and controlled by medical men, with the happiest results to suffering humanity.

The wards of our hospitals, and the similar benevolent enterprises for the relief of our afflicted fellow beings, are attended by medical men, who make their abode amid cries and groans of agony, and the keen sufferings of their afflicted fellow men. There he is to console the suffeng, to raise the drooping and cast down spirits with the soothing whispers of hope, and to give consolation and promise to the sinking and dying. Day by day, he is in communion with sorrow and woe, and hourly dispensing blessings and benefits.

A frightful epidemic with its grim and ghastly features, enters the gates of the populous city—or penetrates into dense settlements and crowded communities. It rages with fearful malignancy, and parents are made childless, children made orphans, and homes made desolate. Deep despair marks the features of every living being, and sorrow and woe fill the minds of the survivors. Where now in these times of trial and general distress, is the physician? The history of all epidemic visitations will furnish the answer. He is abroad, on his mission of mercy, and while others seek safety in flight, he is at his post of duty, faithfully and manfully contending with the deadly foe. Very often, too, he dies in the struggle himself, and follows to the silent tomb, those who were beyond his power to save.

As a great almoner of good, medical science has not been contented with the discharge of those duties, the fortunate tendencies of which experience has made familiar to community and its members. It has been on the road of inquiry for still greater capacities and powers. The atribute of humanity has given direction and an incentive to inquiry, and investigation following, has resulted in the discovery of large additions to the means and appliances of relief. The improvements in surgical skill by which the infliction of unnecessary pain and suffering has been prevented, in very many instances, are triumphs of themselves worthy the great objects to be accomplished. Formerly hot searing irons were used to arrest hemorrhage in capital operations, and caldrons of boiling oil were prepared into which the stump of an amputated limb was immersed, in order to arrest the bleeding. Pare invented the ligatures of silk, which cost no suffering, and were painless in their use, and yet he was at that early period of the history of the world, prosecuted for the innovation upon an old and barbarous custom.

Actuated by humanity, Jenner prosecuted his inquiries into vaccination, the result of which has saved millions of the lives of the human family. Opium has been extolled by the poets as almost a "divinity," because of the repose it affords from suffering and anguish. Chloroform is another potent article, in hushing to sleep the senses and rendering them unconscious to physical distress. It has high claim upon our gratitude, for not only is pain unfelt, but also are the mental terrors, often more severe than physical torture itself, unheeded under surgical operations.

In our cities and municipal organizations, health officers are appointed, and these are usually taken from the ranks of the profession. In the discharge of their duties they are compelled to visit the dens of squalidity and filth, to seek out deleterious agents and elements destructive to public health, and provide for their abatement. Quarantine, and other sanatary regulations, required to protect communities from the invasion and spread of contagious diseases, are dependant upon the profession for their establishment and effectiveness. From an examination of the bills of mortality and by the institution of comparison, it is apparent that in proportion to the number attacked with disease a less number die now than formerly. This fact has been well ascertained; which shows that medical science is, year after year, acquiring more and more control over disease.

But who of all the prefessions or departments of life, save those actually employed in it, have done more for the advancement of the cause of education, than medical men? The wide diffusion of knowledge, by the common school system, has ever received the zealous and efficient support of medical men.

The collateral sciences and the arts, have all drawn liberally from the services of the profession. Geology and mineralogy continue to derive important aid from medical men. Drs. Owen, Shumard, Dean, Hildreth, and others eminent in their profession, have enriched natural science by their labors in this department; and what a fund of sedate and interesting knowledge has been secured by their labors!

Agriculture has been a subject of much labor and interest, and an object of much care by the profession of medicine in all ages. Celsus, an eminent physician in the time of Tiberius, wrote several works on the subject, since which the wisest and best of the profession, have yearly contributed to this important department of the industrial interests of the country. The chemical analysis of the soils and subsoils, the mode and manner in which the different specimens of vegetable productions glean their nutrient particles and appropriate them to their growth and perfection in the several varieties, was most ably discussed by one of the faculty, but a short time since, in this place.

Although Watt conceived the mode by which the use of steam could be made effective, as a propelling power, still the manner of estimating its temperature and elasticity, so essential to fixed results, certainty and safety in its use, was reserved for the triumphant investigation of Drs. Ure, Young and Dalton, and others in the profession.

Medical men have furnished a number of statesmen and patriots. In the halls of legislation they are requisite, in the passage of senatory laws and those involving medico-legal considerations. Dr. Edwards, whilst a member of Congress, obtained the passage of a bill providing for the confiscation of impure drugs, and imposing penalties for their introduction. The consequence is, that a traffic highly injurious to health and life has been arrested, and our country is now supplied with pure and certain medicines.

When we turn back to the history of the past, we indulge in proud reflection and grateful retrospection, to that epoch in the history of our beloved country, which marks the pages relating to our revolution. There we find the names of Rush, Bartlett and Warren, all physicians, who mingled in the councils of that trying period. The latter died valiantly fighting upon the battle field, and a monument erected to the patriot dead—by the gratitude of those who enjoy the blessings for which he shed his blood—now marks the spot where he fell.

Such, then, gentlemen, is the history of medical science, and such its origin. Although cultivated, practiced and propagated by slaves; spurned by men and treated with contumely, because too degrading for even a passing regard, by those of prominence and influence; yet it has risen to its present exalted position, and its history is that of every other great system, having its origin in humility and obscurity. Such too, the benefits it has conferred upon the other sciences and the arts, and like them has struggled into existence under opposing and discouraging circumstances. It has been sanctified by the highest attributes of humanity, characterized by its all-pervading and happy influences upon the well-being of man, and honored and ennobled because of the number, the devotion, the sacrifices, and character of its disciples and votaries. It is with the noble purpose of entering upon its duties as the department of usefulness in this life, which you have selected for yourselves, that you are now here among us. The preparation is scarcely less arduous, or less responsible. In your initiatory steps, you will often falter, will often find yourselves groping in darkness and your pathway obstructed. Patience must be exercised until the gloom be dispelled, and faithfulness and perseverance will remove out of the way opposing barriers. High attainments must be reached for, and by an upward and onward movement; for be assured they will never, unsolicited, descend to you. To attain to the honorable and dignified position to which you aspire in the world, you must labor to deserve it. And yet we would not discourage you. The dark and threatening storm-cloud which overcasts all nature in frightful gloom, often shows a silvery bright light along its margin. It may blacken the heavens for a time with its dark outline, but soon the glories of the sun, which had been temporarily obscured, may pour forth beneath its dark shadows, and reflect upon its sombre face the bright hues of the rainbow of promise. It rests with yourselves, if you desire the honors and rewards of a life devoted to the best interests of humanity. There does not exist that difference which is generally supposed among minds, for subsequent circumstances often modify their destiny, in a good degree, for usefulness and efficiency. Perseverance, energy and action, are more frequently the elements of true greatness, than intuition, mental strength and power.

The history of all great men, as has already been shown, is the tale of toils, labors, disappointments and sacrifices. They have willed;—and it was done,—they have persevered and it was accomplished.

Then let no circumstances discourage, let no difficulty dispirit you let no obstacle arrest your progress. Be true to yourselves and you will be just to the great object for which you came, as well as to those who follow you with their ardent hopes and wishes for your prosperity and success.

The institution in which you have enrolled yourselves for a time as pupils, offers you high inducements. If you studiously apply yourselves you will not go away disappointed in the prospect entertained for improvement. A strict supervisory care will be exercised over your progress, and every effort will be exerted for your advancement. It is under highly favorable auspices and its capacity for usefulness is still on the increase. There is a united harmonious design, as there will be a united effort on the part of your instructors, to lead you on from step to step in the progress of your acquirements, and your best interests will be the constant and earnest study of the faculty.

It is under the parental care of the State Government through its Trustees, and it has the confidence and prayers of the community for its success. Our wise legislators look upon it with the deepest solicitude and interest, because it is intimately incorporated and blended with the well-being of the State. The profession, with a zeal and ardor worthy themselves and their high calling, hope for it, labor for it, and look with pride upon it, as the dispenser of good and the fountain of medical truth and light. And its ALUMNI placed around as sentinels, ever vigilant, and ever active in the exercise of their professional obligations, look back, and send back, their sincere heartfelt prayers for the continued success of their alma mater.

Under such auspices, with such encouragements, with the unanimity which prevails in its councils, with the devotion which every one connected with it displays for its present success and ultimate triumph, it is destined to a high and an elevated position as one of the institutions of the State. And if constant, unwearied effort and zeal ever has or ever will effect aught in any great enterprise, much will be accomplished in this, because of the settled and fixed determination that it shall prove still more worthy of being sustained, and that the profession and the people will enroll it as one of the essential elements of scientifiic advancement in the State. All that is required is that the favoring smile of a generous public and an enlightened profession may be continued to it, and that their protecting ægis will be thrown over it. Then difficulties may surround it, enemies may assail it, and treasonable conspiracies may be formed to crush it, but all will fall harmless at its threshold or reflect back upon the authors of the foul and sacrilegious attempt at the destruction of one of the State's own institutions, and an object of solicitude on the part of the people. It would be vandalism to disturb one single fibril in its youthful structure, lest its healthfulness be interfered with and its capacity for good be impaired.

We would call the attention of the citizens of the city to the large increase over last session. A class, we are free to say without the fear of the charge of adulation, of as much intellectual promise as any other class of the number. They are here from our own State, from other States around us, and some of them from the Eastern States; and nearly all are strangers. We ask for them, that which we are assured they will receive at your hands, that considerate attention which they well deserve. And we would also say to our citizens, as we would say to the people of the State, you have evinced the liveliest interest in the success of this institution, and you have repeatedly given substantial evidence of your regard for it. In this, you have acted intelligenlty, for the benefits substantively, mentally, morally and socially, will be felt by you, and with the growth of the institution, these benefits will multiply. We therefore ask a continuation of your friendship; and we do not as a faculty crave it because they are favors personally conferred upon us, for we derive no profit or emolument from it; but we ask it in the name of medical science, of the profession, and of society at large.

